# Supplementary material for: TNFAIP8 controls murine intestinal stem cell homeostasis and regeneration by regulating microbiome-induced Akt signaling
Source: Nat Commun. 2020 May 22;11:2591. doi: 10.1038/s41467-020-16379-2 (PMC7244529; doi:10.1038/s41467-020-16379-2)
Supplement: Supplementary file 1 — Supplementary Information [file 41467_2020_16379_MOESM1_ESM.pdf]

TNFAIP8 controls murine intestinal stem cell homeostasis and regeneration by regulating microbiome-induced Akt signaling, Goldsmith et al.

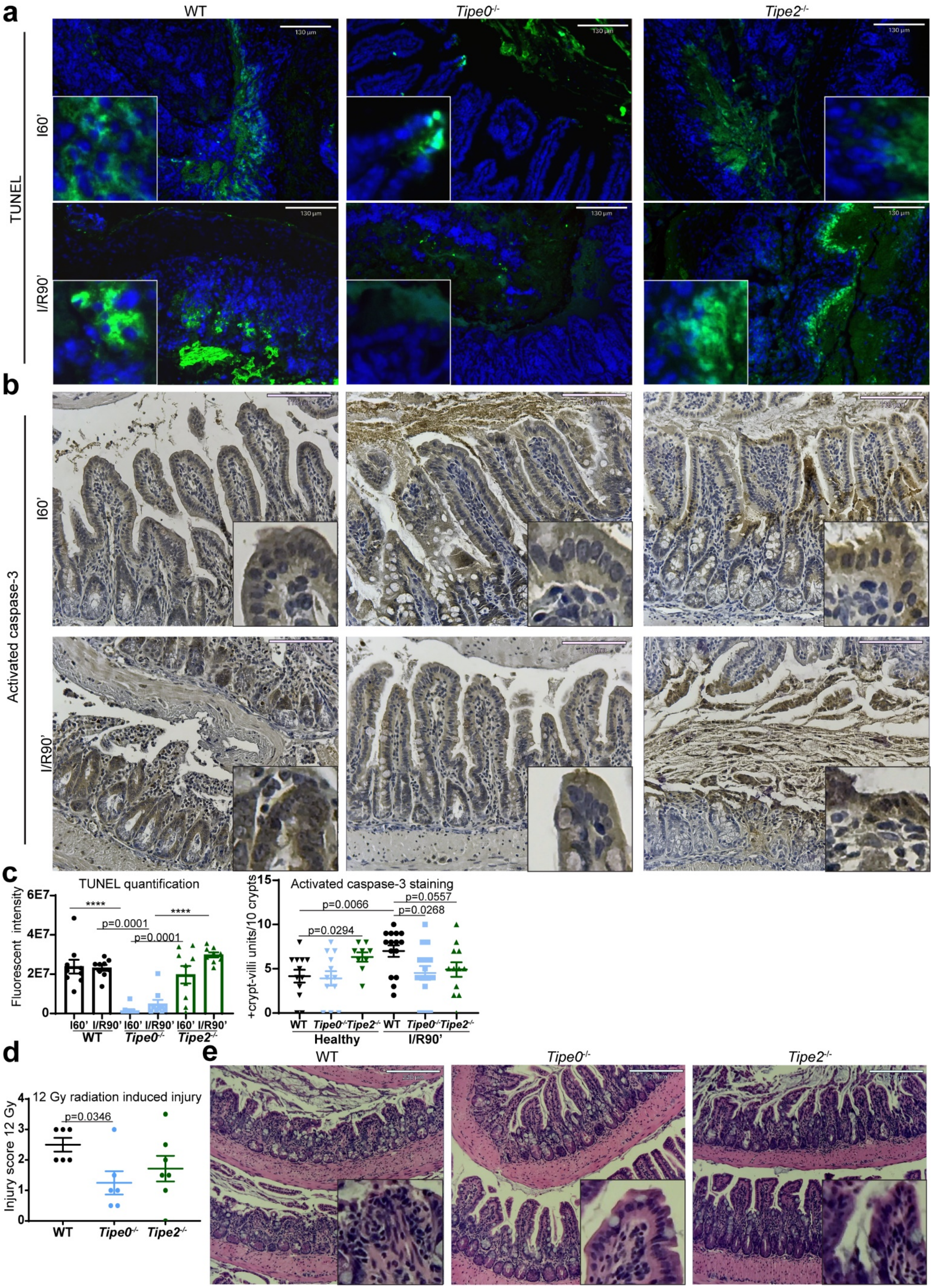

a
WT
Tipe0-/-
Tipe2-/-
TUNEL
I60’
I/R90’
130 μm
b
Activated caspase-3
I60’
I/R90’
c
TUNEL quantification
Fluorescent intensity
6E7
4E7
2E7
0
****
p=0.0001
p=0.0001
****
I60’
I/R90’
WT
Tipe0-/-
Tipe2-/-
Activated caspase-3 staining
+crypt-villi units/10 crypts
15
10
5
0
p=0.0557
p=0.0066
p=0.0268
p=0.0294
WT
Tipe0-/-
Tipe2-/-
Healthy
I/R90’
d
12 Gy radiation induced injury
Injury score 12 Gy
4
3
2
1
0
p=0.0346
WT
Tipe0-/-
Tipe2-/-
e
WT
Tipe0-/-
Tipe2-/-

Supplementary Figure 1: Loss of TIPE0, but not TIPE2, results in decreased apoptosis during the ischemic injury and protection from radiation-induced injury. A) TUNEL assay of tissue subjected to 60 min of ischemia (I60') or 60 min of ischemia followed by 90 min of reperfusion (I/R90'). Images representative of 3/3 mice/group. Green=TUNEL signal, blue=DAPI counterstain; bars=130 μM; inserts 4X magnified. Controls in Supplementary Figure 2a. B) Activated caspase-3 staining in healthy and I/R90' samples. Images representative of 3 images from 3-5 mice/group, as elaborated in (C). Bars=110 μm; inserts 4X magnified. C) Quantification of IF/IHC from (A&B). 3 images per slide were selected at random and quantified; TUNEL (A) was quantified by total green fluorescent intensity; N=9 image/group (3 images each from 3 mice/group); analysis by one-way ANOVA with Tukey' multiple comparison test. Activated caspase-3 staining (B) was quantified by counting crypt-villi units with 50% or more positive staining cells per 10 units; N as follows: for WT, *Tipe0*$^{-/-}$, and *Tipe2*$^{-/-}$ I/R90', 3 images each from 4 mice/group for N=12; for WT I/R90' and *Tipe0*$^{-/-}$ I/R90', 3 images each from 5 mice/group for N=15; for *Tipe2*$^{-/-}$ I/R90', 3 images each from 3 mice for N=9. Multiple group comparisons were by Kruskal-Wallis one-way ANOVA. Two-tailed Mann-Whitney U test was used to confirm ANOVA findings. D-E) Data from 2 days post-12 gy radiation. D) Histological score and E) representative mean images of N=6 mice/group (7 for *Tipe2*$^{-/-}$) are shown; bars=220 μm, inserts 4X magnified. Analysis by two-tailed Mann-Whitney U test. For all graphs, ****, p<0.0001, or as indicated; error bars show mean±SEM. Source data are provided as a source data file.

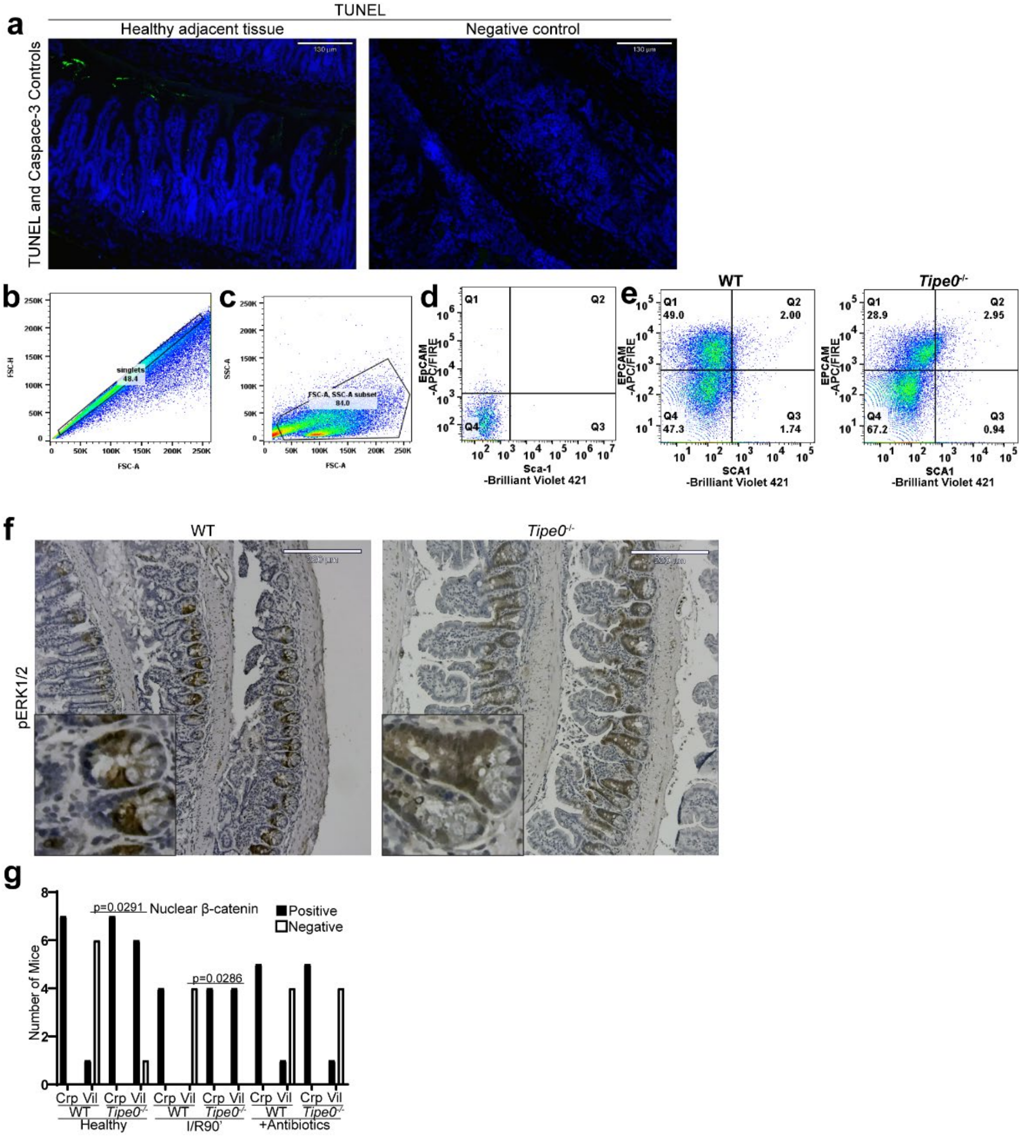

Supplementary Figure 2: Sample gating for flow cytometry and supplemental IHC/IF controls/analyzes. A) Sample control images from healthy tissue adjacent to ischemic tissue on the same swiss roll and negative staining control. Images representative of 3/3 mice/group. Green=TUNEL signal, Blue=DAPI counterstain; bars=130 μM; inserts 4X magnified. B-E) All flow experiments were independently repeated two times in triplicate. B) Doublets were removed from total population using FSC-A and FSC-H. C) Total singlets were gated using FSC-A and SSC-A. D) Isotype controls were used to determine the background caused by nonspecific antibody binding. E) Sca-1 and EpCAM positive population were gated based on isotype controls staining. F) IHC for pERK1/2; bars=220 μm, inserts 4x magnified, images representative of 3 mice/group. G) Nuclear β-catenin staining from Figure 9b was determined to be positive if 50% or more of the positive staining cells had nuclear staining. Crp=Crypt; Vil=Villi for the nuclear staining. P as indicated, with analysis by two-sided Fisher's exact test; error bars show mean±SEM. N=7 for all healthy mice, N=4 for all ischemic mice, N=5 for all mice given antibiotics.

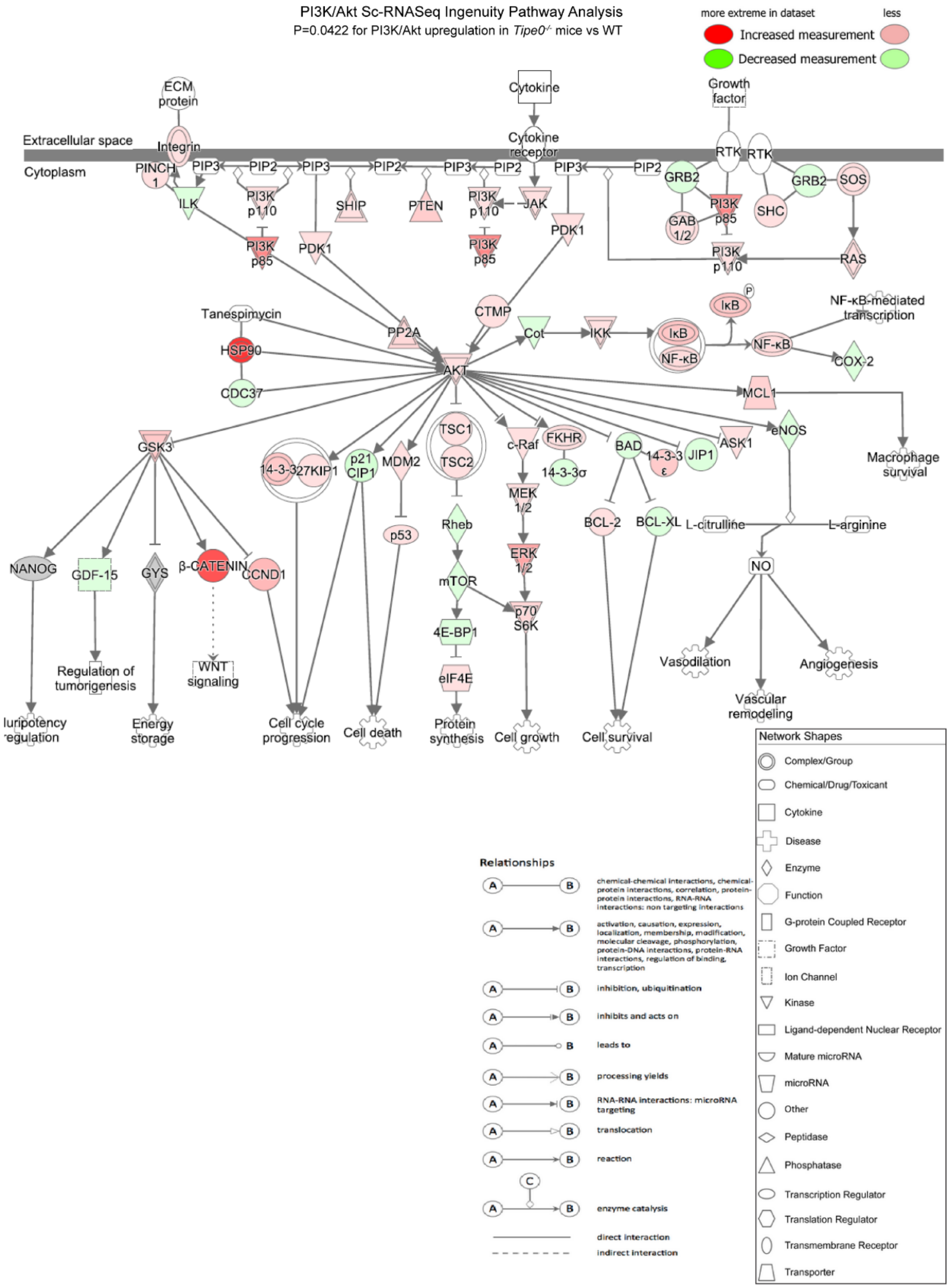

Supplementary Figure 3: Ingenuity Pathway analysis of the Sc-RNASeq data for PI3K/Akt signaling. Graphical pathway heat mapped to show increased measurements in red and decreased measurements in green. For this network, p=0.0422 by right-tailed Fisher's exact test for upregulation of total PI3K/Akt associated signaling in the knockout.

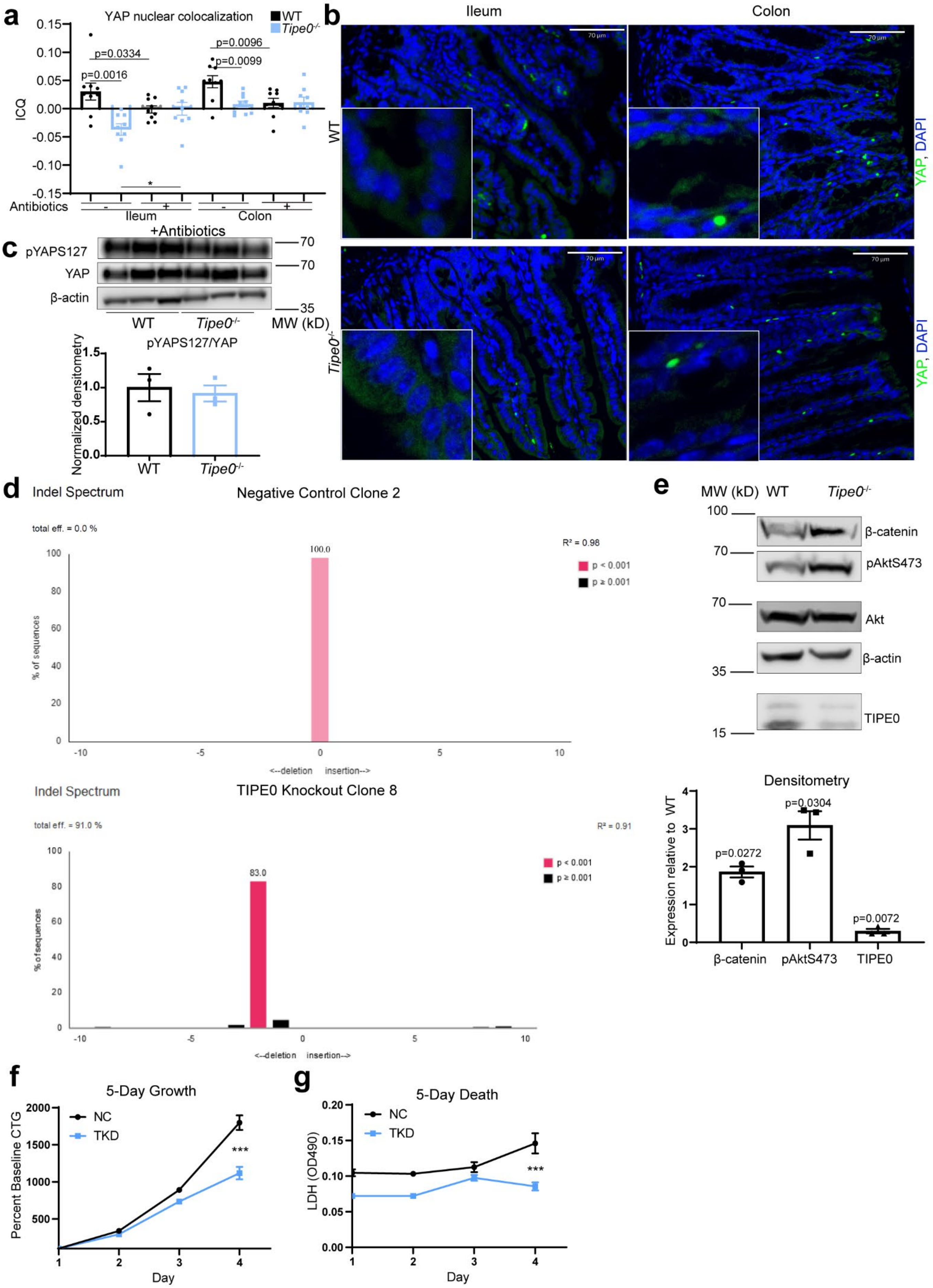

a
YAP nuclear colocalization
WT
Tipe0-/-
ICQ
p=0.0016
p=0.0334
p=0.0096
p=0.0099
*
Antibiotics
Ileum
Colon
b
Ileum
Colon
WT
Tipe0-/-
YAP, DAPI
70 μm
+Antibiotics
c
pYAPS127
YAP
β-actin
MW (kD)
pYAPS127/YAP
Normalized densitometry
d
Indel Spectrum
Negative Control Clone 2
total eff. = 0.0 %
R² = 0.98
p < 0.001
p ≥ 0.001
% of sequences
<--deletion insertion-->
TIPE0 Knockout Clone 8
total eff. = 91.0 %
R² = 0.91
e
MW (kD) WT Tipe0-/-
β-catenin
pAktS473
Akt
β-actin
TIPE0
Densitometry
Expression relative to WT
p=0.0272
p=0.0304
p=0.0072
f
5-Day Growth
NC
TKD
Percent Baseline CTG
Day
***
g
5-Day Death
LDH (OD490)

Supplementary Figure 4: Antibiotics ablate changes in YAP signaling seen with loss of TIPE0 and generation and characterization of Tipe0-knockdown (TKD) CMT-93 cells via CRISPR/Cas9. A-B) YAP nuclear co-localization (A) by intensity correlation quotient (ICQ) in healthy ileums and colons of mice subjected to antibiotics to ablate the microbiota. Data from Figure 5h-i was generated at the same time and is repeated here for comparison. N=9/group (3 mice/group, with 3 images/mouse analyzed). Multiple group comparisons were by Kruskal-Wallis one-way ANOVA. Two-tailed Mann-Whitney U test was used to confirm ANOVA findings. (B) Representative images from (A); bars=70 μm; inserts are 4X magnified. C) Western blot to assess for pYAPS127/YAP ratio in freshly isolated enterocytes from mice given antibiotics to ablate their microbiota. Densitometry analysis below, relative to WT with β-actin as the loading control; N=3 mice/group; p=. D) Sample TIDE indel analysis of negative control and TIPE0 knockout clones; analysis by two-tailed t-test. E) Western blot of final pooled negative control (NC) and Tipe0-knockout (TKD) clones, showing that the pooled TIPE0-knockout clones are a stable knockdown, and that pAktS473 signaling and downstream β-catenin levels have changes similar to what is seen in $Tipe0^{-/-}$ enterocytes. Densitometry is pooled from 3 different passages of cells over a 3-week period of time. Analysis by one-sample two-tailed T-test. F) 5-day growth assay, as measured by CTG, comparing NC and TKD CMT-93 cells; p=0.0001. G) 5-day death, as measured by LDH accumulation, analyzing the supernatant from the cells of (F); p=0.0002. For F&G, data representative of 2 independent experiments, N=5/group, analysis by 2-way ANOVA. For all graphs error bars show mean±SEM; * p<0.05; ** p<0.01; ***p<0.001; ****, p<0.0001. Source data are provided as a source data file.
