# Supplementary material for: TNFAIP8 controls murine intestinal stem cell homeostasis and regeneration by regulating microbiome-induced Akt signaling
Source: Nat Commun. 2020 May 22;11:2591. doi: 10.1038/s41467-020-16379-2 (PMC7244529; doi:10.1038/s41467-020-16379-2)
Supplement: Supplementary file 5 — Reporting Summary [file 41467_2020_16379_MOESM5_ESM.pdf]

nature research

Corresponding author(s): Jason R Goldsmith
Youhai Chen

Reporting Summary

Nature Research wishes to improve the reproducibility of the work that we publish. This form provides structure for consistency and transparency in reporting. For further information on Nature Research policies, see Authors & Referees and the Editorial Policy Checklist.

Statistical parameters

When statistical analyses are reported, confirm that the following items are present in the relevant location (e.g. figure legend, table legend, main text, or Methods section).

n/a	Confirmed	
☐	☒	The exact sample size (n) for each experimental group/condition, given as a discrete number and unit of measurement
☐	☒	An indication of whether measurements were taken from distinct samples or whether the same sample was measured repeatedly
☐	☒	The statistical test(s) used AND whether they are one- or two-sided<br>*Only common tests should be described solely by name; describe more complex techniques in the Methods section.*
☐	☒	A description of all covariates tested
☐	☒	A description of any assumptions or corrections, such as tests of normality and adjustment for multiple comparisons
☐	☒	A full description of the statistics including central tendency (e.g. means) or other basic estimates (e.g. regression coefficient) AND variation (e.g. standard deviation) or associated estimates of uncertainty (e.g. confidence intervals)
☐	☒	For null hypothesis testing, the test statistic (e.g. F, t, r) with confidence intervals, effect sizes, degrees of freedom and P value noted<br>*Give P values as exact values whenever suitable.*
☒	☐	For Bayesian analysis, information on the choice of priors and Markov chain Monte Carlo settings
☒	☐	For hierarchical and complex designs, identification of the appropriate level for tests and full reporting of outcomes
☒	☐	Estimates of effect sizes (e.g. Cohen's d, Pearson's r), indicating how they were calculated
☐	☒	Clearly defined error bars<br>*State explicitly what error bars represent (e.g. SD, SE, CI)*

Our web collection on statistics for biologists may be useful.

Software and code

Policy information about availability of computer code

Data collection	no software used
Data analysis	Open source code for the R-suite was used for RNA-Seq analysis, as described within the methods section. For sc-RNASeq, analysis, initial data processing of samples was performed using Cellranger (v.2.1.0, 10x Genomics). Cellranger mkfastq was used to generate demultiplexed FASTQ files from the raw sequencing data. Next, cellranger count was used to align sequencing reads to the mouse reference (mm10), and generate single cell gene barcode matrices. Cellranger aggr was used to aggregate the matrices and normalize by mapped read depth to account for sequencing depth. Post processing and secondary analysis of the aggregated dataset was performed using the Seurat package (v.3.0) within R (v.3.5.1). Ingenuity Pathway Analysis (Qiagen) was also used. ImageJ with FIJI (NIH) was used for image analysis.

For manuscripts utilizing custom algorithms or software that are central to the research but not yet described in published literature, software must be made available to editors/reviewers upon request. We strongly encourage code deposition in a community repository (e.g. GitHub). See the Nature Research guidelines for submitting code & software for further information.

Data

Policy information about availability of data

All manuscripts must include a data availability statement. This statement should provide the following information, where applicable:

- Accession codes, unique identifiers, or web links for publicly available datasets
- A list of figures that have associated raw data
- A description of any restrictions on data availability

The RNA-Seq datasets generated during and/or analyzed during the current study will be deposited in GEO at the time of manuscript acceptance, with accession codes as follows: XXX. Tables S1 and S2 have the associated data used in the manuscript. There are no restrictions on data availability.

Field-specific reporting

Please select the best fit for your research. If you are not sure, read the appropriate sections before making your selection.

☒ Life sciences ☐ Behavioural & social sciences ☐ Ecological, evolutionary & environmental sciences

For a reference copy of the document with all sections, see nature.com/authors/policies/ReportingSummary-flat.pdf

Life sciences study design

All studies must disclose on these points even when the disclosure is negative.

Sample size	An N of 6 per biological experiment was chosen based on prior work with ischemia/reperfusion, where this sample size was determined based on power calculations to detect a histological score change of 1 with a variance of 0.5 within a group. This was extended to all animal models here for consitency.
Data exclusions	Ischemia data was excluded when a mouse showed zero signs histologically and by gross observation of ischemic injury. This rarely happens when a clamp fails to hold and is obvious by gross and histological pathology. Other data were excluded if they fell outside of 3 SD of the mean of their group, with them included in that group for that analysis.
Replication	All experiments were repeated on different days. At least two different biological replicates were performed for experiments derived from primary cells, and often many more.
Randomization	All mice used were age and gender matched, and were otherwise randomly selected.
Blinding	Investigators were blinded during all histological analyses. Blinding in other samples was not possible, as all primary tissues from which subsequent samples were derived were labeled by genotype.

Reporting for specific materials, systems and methods

Materials & experimental systems

n/a	Involved in the study	
☒	☐	Unique biological materials
☐	☒	Antibodies
☐	☒	Eukaryotic cell lines
☒	☐	Palaeontology
☐	☒	Animals and other organisms
☒	☐	Human research participants

Methods

n/a	Involved in the study	
☒	☐	ChIP-seq
☐	☒	Flow cytometry
☒	☐	MRI-based neuroimaging

Antibodies

Antibodies used	Primary Antibodies: anti-Sca-1 clone E13-161.7 (122502, LotB253354, Biolegend); Biotin anti-Brdu MoBU-1 (317904, lot B254434, Biolegend); anti-CD11b at 1:200 (NB110-89474, Novus Biologicals); anti-E-cadherin mouse mAb (14472, lot 5, Cell Signaling); anti-β-catenin rabbit mAB (8480, lot 5, Cell Signaling); anti-YAP rabbit mAb (14074, lot 4, Cell Signaling); anti-CD45.1 anti-mouse PE-Cy7 Clone A2 (Lot E07571-1633, eBiosciences); anti-CD45.2 anti-mouse APC Clone 104 (lot 4290824, eBiosciences); anti-pGSK3βS9 rabbit mAb (9323, Lot 15, Cell Signaling); anti-pAKTS473 rabbit mAB (4060, lots 19 & 23, Cell Signaling); anti-activated-Caspase-3 (AF835, lot CFZ3416111, R&D Systems); anti-Ki67 (ab16667, lot GR289011-6, Abcam); anti-human lysozyme EC 3.2.1.17 (A0099, lot 20040597, Agilent); anti-human Keratin-20 (13063, lot 1, Cell Signaling); Phalloidin-555 (8953, Cell Signaling); anti-mouse β-actin (3700, lot 16,Cell Signaling); anti-Rictor antibody (sc-271081, Santa Cruz); custom anti-TNFAIP8 rabbit polyclonal IgG (15790-1-AP,Proteintech); anti-Cd326 Ep-CAM APC/FIRE (118229, Biolegend); anti Ly-6A/E (d7) Brilliant Violet 421 (108127, Biolegend)<br>Secondary antibodies: Anti-rabbit 488 IgG Fab2 (4412, Lot 19, Cell Signal); Anti-rat 488 IgG Fab2 (4416, lot 13, Cell Signal); Anti-mouse 555 IgG Fab2 (4409, Lot 16, Cell Signal); VectaKit Elite ABC, anti-rabbit and anti-mouse (Vector Labs); Alexa Fluor®488 streptavidin (016-540-984, Lot 138230, Jackson ImmunoResearch); Alexa Fluor® 790 AffiniPure Goat Anti-Rabbit IgG (H+L) (111-655-144, Jackson ImmunoResearch)
Validation	Sca-1 antibody was previously validated by Dr. Ophir Klein and discussed in private communications as his recent Nature, 2018 paper. Anti-Brdu was previously validated in the literature and discussed in private communications with Dr. Jerry Turner. Anti-CD11b was validated by literature search. Anti-E-caderin was validated by manufacturer provided citations and literature search. Anti-B-catenin was validated by prior personal use and manufacturer provided citations. Anti-YAP was validated by Western and by manufacturer provided citations. The anti-CD45.1 antibodies were previously validated in the lab by flow cytometry of immune cells gathered from mice of each CD45-isotype; anti-pGSK3βS9 and anti-pAKTS473 antibodies were validated by prior use in the lab and manufacturer provided citations. Anti-Ki67 was validated by literature review and comparison to known ki-67 staining patterns. Anti-activated-Caspase-3 was validated by manufacturer provided citations. Anti-human lysozyme and anti-keratin-20 were validated by an enteroid-specific IF protocol publication to cross-react in mice and this was confirmed by the staining pattern observed. Phalloidin-555 was validated based on manufacturer provided citations and prior use in the lab. β-actin was validated based on numerous prior references using this antibody. anti-Rictor was validated based on manufacturer provided citations; anti-TNFAIP8 was validated using purified protein and knockout mouse tissue in our lab.<br>Fluorescent secondary antibodies from Cell Signal and Jackson ImmunoResearch were validated by prior use in our lab, as well as manufacturer data sheets with listed publications. VectaKit Elite ABC kits were validated by prior lab use and manufacturer data sheets with listed publications. Streptavidin conjugate was recommended by Dr. Jerry Turner who previously validated the antibody.

Eukaryotic cell lines

Policy information about cell lines

Cell line source(s)	CMT-93, ATCC® CCL-223™
Authentication	Cell lines were authenticated based on gross morphology, which is distinctive, and verified by sequencing to be colonic and polyploid.
Mycoplasma contamination	Cells were certified free of mycoplasma contamination when they were received.
Commonly misidentified lines (See ICLAC register)	N/A

Animals and other organisms

Policy information about studies involving animals; ARRIVE guidelines recommended for reporting animal research

Laboratory animals	C57BL/6 mice; strains: Wild type, TNFAIP8-/-, TNFAIP8L2-/-, and
Wild animals	N/A
Field-collected samples	N/A

Flow Cytometry

Plots

Confirm that:

- [x] The axis labels state the marker and fluorochrome used (e.g. CD4-FITC).
- [x] The axis scales are clearly visible. Include numbers along axes only for bottom left plot of group (a 'group' is an analysis of identical markers).
- [x] All plots are contour plots with outliers or pseudocolor plots.
- [x] A numerical value for number of cells or percentage (with statistics) is provided.

Methodology

Sample preparation	Enterocytes were isolated as above and then fixed in fresh, ice-cold 1% PFA/PBS for 10 minutes. Cells were then washed twice with 2% FBS in PBS, then digested in a 10 ml PBS solution containing 2% FBS and 0.25 mg/ml collagenase IV for 30 minutes at 37 under shaking (200 rpm). Digested cells washed twice with 2% FBS and 2mM EDTA in PBS, passed through 70 µM filter to collect single cells. Cells were stained 1:100 dilutions at RT for 20 min before washing. Isotype controls were used at 1:100 dilutions to determine the background caused by nonspecific staining.
Instrument	LSRII (BD Biosciences, San Jose, CA)
Software	FlowJo v10.0.7 (BD)
Cell population abundance	Abundance of the cells in question were relatively low, in the single-digit percentages, in line with prior work. Contamination was excluded by looking at EpCAM+ cells to select for enterocytes, followed by Sca-1 to look at this marker in question.
Gating strategy	Doublets were removed from total population using FSC-A and FSC-H. Total singlets were gated using FSC-A and SSC-A. Isotype controls were used to determine the background caused by nonspecific antibody binding. Sca-1 and EpCAM positive population were gated based on isotype controls staining.

- [x] Tick this box to confirm that a figure exemplifying the gating strategy is provided in the Supplementary Information.
